# BCG vaccination in southern rural Mozambique: an overview of coverage and its determinants based on data from the demographic and health surveillance system in the district of Manhiça

**DOI:** 10.1186/s12887-018-1003-4

**Published:** 2018-02-13

**Authors:** Elena Marbán-Castro, Charfudin Sacoor, Ariel Nhacolo, Orvalho Augusto, Edgar Jamisse, Elisa López-Varela, Aina Casellas, John J. Aponte, Quique Bassat, Betuel Sigauque, Eusebio Macete, Alberto L. Garcia-Basteiro

**Affiliations:** 1 ISGlobal, Barcelona Ctr. Int. Health Res. (CRESIB), Hospital Clínic-Universitat de Barcelona, C/Rosselló 132, 08036 Barcelona, Spain; 2 Centro de Investigação em Saúde da Manhiça (CISM), Rua 12, Vila de Manhiça, CP 1929 Maputo, Mozambique; 3 ICREA, Pg. Lluís Companys 23, 08010 Barcelona, Spain; 4 Amsterdam Institute for Global Health and Development (AIGHD), Amsterdam, The Netherlands

**Keywords:** BCG vaccine, Tuberculosis, Pediatrics, Expanded programme on immunization, Epidemiology, Mozambique

## Abstract

**Background:**

Over the past four decades, the World Health Organization established the Expanded Programme on Immunization (EPI) to foster universal access to all relevant vaccines for all children at risk. The success of this program has been undeniable, but requires periodic monitoring to ensure that coverage rates remain high. The aim of this study was to measure the BCG vaccination coverage in Manhiça district, a high TB burden rural area of Southern Mozambique and to investigate factors that may be associated with BCG vaccination.

**Methods:**

We used data from the Health and Demographic Surveillance System (HDSS) run by the Manhiça Health Research Centre (CISM) in the district of Manhiça. A questionnaire was added in the annual HDSS round visits to retrospectively collect the vaccination history of children under the age of 3 years. Vaccinations are registered in the National Health Cards which are universally distributed at birth. This information was collected for children born from 2011 to 2014. Data on whether a child was vaccinated for BCG were collected from these National Health Cards and/or BCG scar assessment.

**Results:**

A total of 10,875 number of children were eligible for the study and 7903 presented the health card. BCG coverage was 97.4% for children holding a health card. A BCG-compatible scar was observed in 99.0% of all children and in 99.6% of children with recorded BCG in the card. A total of 93.4% of children had been vaccinated with BCG within their first 28 days of life. None of the factors analysed were found to be associated with lack of BCG vaccination except for living in the municipality of Maluana compared to living in the municipality of Manhiça; (OR = 1.89, 95% CI: 1.18-3.00). Coverage for other EPI vaccines during the first year of life was similarly high, but decreased for subsequent doses.

**Conclusions:**

BCG coverage is high and timely administered. Almost all vaccinated infants develop scar, which is a useful proxy for monitoring BCG vaccine implementation.

**Electronic supplementary material:**

The online version of this article (10.1186/s12887-018-1003-4) contains supplementary material, which is available to authorized users.

## Background

Tuberculosis (TB) remains a global public health concern, responsible for an estimated 1.8 million deaths in 2015. It stands as the leading cause of death by an infectious agent worldwide [1]. The only available vaccine to fight TB is the Bacille Calmette-Guérin (BCG) vaccine, first administered in 1921 and, probably the most widely used vaccine in the world [2, 3]. Although the efficacy of BCG against pulmonary TB has been questioned [4], it remains an essential approach for prevention of the most severe forms of TB in children (with an estimated efficacy against miliary TB and TB meningitis of 77 and 73% respectively) [5, 6]. It also reduces infection [7] and all-cause mortality through non-specific effects of the immune system [8]. A recent study has shown a long-lasting protection of BCG, being more cost-effective than previously thought [9]. Moreover, non TB beneficial effects have been reported such as protection against other causes of death, or reduced risk of death from pneumonia and malaria (studies from African and Asian low-income countries) [2, 10, 11]. Administered at birth, BCG reduces neonatal mortality by 48% in low-birth weight infants [12]. An added importance of BCG is its proximity to the delivery event and thus being the entry point to EPI and other health packages [12].

The WHO recommends vaccinating all newborns in endemic areas with BCG at birth, except in cases of positive or suspicion of HIV infection [4]. In settings where HIV status cannot be discarded at the time of vaccination, for example, infants born to HIV-positive mothers with unknown status and lacking suggestive symptoms, BCG should be given after considering local epidemiology.

The development of a scar secondary to BCG vaccination is a good indicator of vaccination response, associated with reduction of childhood mortality [5, 11, 13], but there are other factors involved in the lack of the immune response, such as cold chain management. The most widely used strategies to assess BCG vaccination include the verification of its administration through vaccination cards [2, 14–19] and the direct observation of a BCG-compatible scar [2, 14, 20, 21]. Studies have reported many risk factors associated with no vaccination, including female gender, great number of siblings, lower mother’s education, low knowledge of vaccine schedule, single or divorced marital status, poor wealth index and low density of health workers, among others [14, 17, 21–24].

Mozambique is one of the countries with highest TB incidence and lowest TB case detection rates in the world [1, 25]. A recent study showed that TB is associated with 6.5% of all deaths in a rural district in the south of the country [26]. TB control strategies are based on improving and enhancing access to diagnosis treatment, and prevention through vaccination or preventive treatment. The Mozambican Expanded Programme on Immunization (EPI) was first introduced in 1979 with a commitment of reducing infant mortality and morbidity by immunization [27]. Nevertheless, constraints related to its weak performance have been identified at several levels: poor programme data management, inadequate logistic, insufficient financial resources and cold chain management, among others [27]. A complete immunization program for the first year of life includes BCG and an Oral Polio Vaccine (OPV) at birth, three more doses of OPV and three doses of pentavalent vaccine (Diphtheria, Tetanus, Pertussis, Hepatitis B, *Haemophilus influenzae* type b) at 6, 10 and 14 weeks, and a measles vaccine at month 9 respectively. More recently, the conjugate vaccines against pneumococcal disease (2009) and against rotavirus (2011) have also been added to this schedule. Vaccines are administered free of charge and at several peripheral health care centres, widening the possibilities of being vaccinated.

The WHO (2015) reports an official estimated BCG coverage for Mozambique of 95% based on data from the Demographic and Health Survey [28]. However, the reliability of these official estimates has been questioned because BCG vaccination coverage differs from institution to institution and estimates have been reported above 100% [18, 29]. Moreover, critical BCG vaccine shortages have been reported between 2013 to 2015 in many countries [30]. Thus, this study was conducted to measure BCG vaccination coverage among children below 36 months of age, through BCG recorded in national health cards and by BCG scar assessment. As secondary objectives, we aimed to a) analyse BCG timeliness, in order to evaluate whether the vaccine was given in the right time period b) compare the coverage of BCG to other vaccines and c) identify the socio-demographic factors that might be associated with lack of BCG vaccination.

## Methods

### Study design and setting

The study was conducted in the district of Manhiça, Maputo Province, a rural area of Southern Mozambique, where the Manhiça Health Research Centre (CISM) runs a Health and Demographic Surveillance System (HDSS) since its foundation in 1996 [31]. It is a high TB and HIV burden area [32, 33]. In 2014, the HDSS was expanded to cover the entire district, an area of 2380 km^2^ that comprises around 38,000 enumerated and geo-positioned households, and about 178,000 individuals. Compared to the official census, DHS, health service data and civil registrations, the HDSS is considered a gold standard tool for population indicators and cross-national comparisons [1, 34].

In Mozambique, where high pediatric TB rates and low case detection rates have been reported [35, 36], children receive a national health card (also called “vaccination card”) at birth or in their first contact with the health system, where immunization, anthropometric and basic health data are registered. All children born in the district of Manhiça participate in the HDSS.

### Design / participants

In every HDSS round, demographic information about births, deaths and migration is updated. This is a cross-sectional study performed at the time of the HDSS census rounds of 2014 and 2015, which included a specific form to collect information about vaccination status. In each round, information was collected for children who were up to 3 years of age, thus in the round of 2014, children born in 2011, 2012 and 2013 were evaluated; and from 2012 onwards for the round of 2015. Information for all children who were less than 36 months of age at the HDSS census rounds was selected. Health cards, whenever available, were evaluated by the field worker, who collected information about administration of all vaccines. In order to estimate BCG and other EPI vaccines coverage through the assessment of vaccination card, we only included children who presented the card at the time of the interview; in order to assess BCG vaccination coverage through the presence of scar, we included all children observed at the visits.

### Data collection and analysis

Data cleaning, prior to data analysis, included deletion of duplicated records or incomplete variables. Duplicated observations occurred because the questionnaire was administered to every child irrespective of having or not responded to previous rounds. This allowed to have the most updated information for missed children in previous visits and newborns. When duplicate observations were present, those observations with the most complete data for all variables were preserved.

BCG vaccination coverage (VC) was defined as the proportion of children with recorded BCG vaccine in their health card divided among children whose health card was assessed and readable. VC was calculated as a proportion of children receiving a BCG or other EPI vaccines divided by the total number of eligible children (those who should have received it according to their age at the time of the visit and whose health card was assessed, readable and without missing dates). VC was calculated as a proportion, with 95% confidence intervals (CI). Information about children included variables such as sex, number of siblings, season of birth and area of residence. Mothers’ data was obtained from other HDSS questionnaires in which information about family members is routinely collected, including religion, education or marital status. Variables at household level, such as wealth index and distance to nearest health centre were also included. The variable wealth index was estimated using principal component analysis (PCA) with variables related to the household assets following the recommendations of Vyas et al. [37].

To measure the coverage of BCG through scar assessment, the number of children who presented a BCG scar was divided by the total number of children assessed for scarring. The coverage was also measured among children with and without health cards and among children with BCG according to their health card. Delay in BCG administration was defined as a child receiving BCG vaccine after the first 28 days of life.

In the descriptive analysis absolute and relative frequencies were calculated. The description included qualitative variables and quantitative variables categorized according to the objective of the study.

Every variable which a priori seemed to be potentially associated with absence of BCG vaccination in the card was tabulated against BCG administration. Odds Ratios with a 95% CI and *p*-values were calculated. A stepwise procedure was carried out in order to build a multivariate logistic regression model using those variables with p-values < 0.15 in the univariate analysis.

The analysis was conducted using Stata 13 (StataCorp LP, College Station, TX, USA). Graphs and tables were produced with Excel (Microsoft Office 2016, USA).

## Results

### Population and socio-demographic characteristics

According to CISM’s HDSS database, 11,537 children were born between 1st January 2011 and 31st December 2014 in the district of Manhiça. From the 10,875 eligible children (born in that period and under 36 months at the time of annual visits), 9512 children were visited. Around 72.9% (7903/10,875) of children presented a health card to the field workers for transcription of the information on vaccination. Of 2972 children whose card was not available, 48.9% of cases declared the reason was that the adult responding to the HDSS questions could not find the card and, in almost a quarter, 23.1%, no reason was recorded.

### BCG and other vaccines coverage

**Fig. 1:**
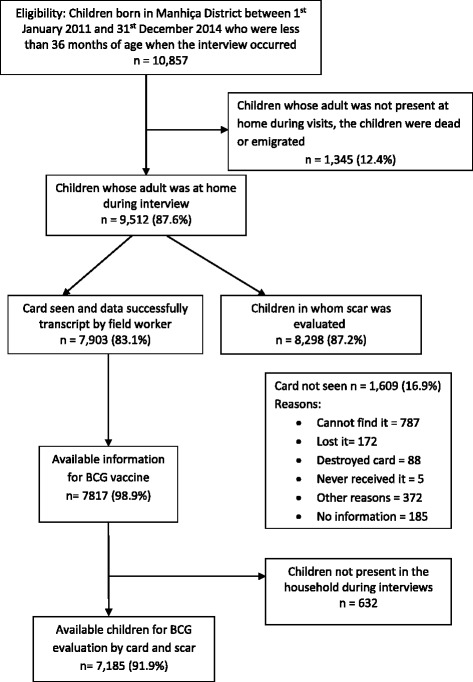
Flow of study participants. Children less than 36 months of age born in Manhiça from 2011 to 2014 and eligible to participate in the study: an adult was at home when interviews occurred, they presented the child’s health card and the children were alive

Among children with a health card, information about BCG vaccination (either yes or no) was recorded in 98.9% of the cases and, from those, 91.9% were present at the time of the interview allowing the evaluation of their arm to see the scar post BCG vaccination (see Fig. 1). Regardless of having the vaccination card, 8298 children were evaluated for presence of BCG scar. Characteristics of study participants are described in Table 1. Additional file 1 presents the characteristics of infants with and without health card.

**Table 1:** Demographic and socioeconomic characteristics of less than 36-months old children

Variable^a^	*n*	%	Variable	*n*	%
Sex			Mother’s antenatal visits		
Boy	3979	50.4	1 to 2	170	9.8
Girl	3921	49.6	3 or more	1573	90.3
Number of siblings			Place of delivery		
None	9	0.5	Health centre	1665	95.6
1 to 2	851	48.8	Home/Way to hospital	77	4.4
3 or above	883	50.7	Type of birth		
Season of birth			Natural	1655	95.0
Rainy	4329	54.8	C-Section	88	5.0
Dry	3574	45.2	Distance to health centre		
Wealth Index			Less than 5 km	988	19.8
1st Quintile	1312	18.4	More than 5 km	3994	80.2
2nd Quintile	1478	20.8	Mother’s marital status		
3rd Quintile	1450	20.4	Single	513	10.1
4th Quintile	1451	20.4	Married/Union	3909	77.2
5th Quintile	1418	20.0	Divorced/Separated	641	12.7
Area			Mother’s education		
Manhiça Sede	1706	21.6	No education	2110	43.0
3 de Fevereiro	1593	20.2	Primary	2308	47.0
Ilha Josina Machel	144	1.8	Secondary or Higher	491	10.0
Xinavane	2215	28.0	Mother’s religion		
Maluana	1689	21.4	Christian	2126	44.5
Calanga	556	7.0	Muslim	42	0.9
			Traditional African	2176	45.5
			Others	435	9.1

^a^ Many variables presented missing data due to lack of completeness of the questionnaire, or because some of them were implemented in different years

**Fig. 2:**
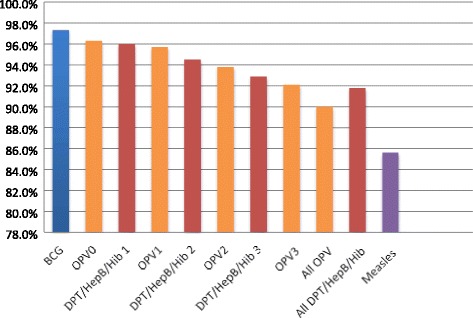
Vaccination coverage among less than 36-month old children in the district of Manhiça (2011-2014)

A total of 7612 children under the age of 36 months whose national health card was evaluated were BCG vaccinated in the district of Manhiça, yielding a BCG coverage of 97.4%. Table 2 and Fig. 2 show the vaccination coverage for all EPI vaccines administered in the district of Manhiça in the first year of life during the years 2011 to 2014. Coverage for each of the four doses of Oral Polio Vaccines were: 96.3%, 95.6%, 93.8% and 92.1%. For the pentavalent DPT/HepB/Hib vaccine, coverage was 96%, 94.5% and 93%. Measles vaccine was received around month 9 of life by 85.6% of infants. Around 90.2% of all study children had received all four doses of Oral Polio Vaccine and 91.8% of the doses of the pentavalent vaccine DPT/HepB/Hib. We found no differences in coverage for any of the vaccines by year of vaccination.

**Table 2:** Vaccination coverage among children aged less than 36 months in the district of Manhiça (2011-2014)

Name of the vaccine	Number of children vaccinated (by card)	Number of children not vaccinated (by card)	Total children evaluated for each vaccine^b^	%	95% CI
BCG	7613	204	7817	97.4%	(95.20, 99.59)
OPV0	7505	289	7794	96.3%	(94.17, 98.54)
DPT/HepB/Hib 1	7466	311	7777	96.0%	(93.83, 98.20)
OPV1	7434	338	7772	95.7%	(93.52, 97.88)
DPT/HepB/Hib 2	7336	425	7761	94.5%	(92.37, 96.71)
OPV2	7274	477	7751	93.8%	(91.70, 96.03)
DPT/HepB/Hib 3	7195	546	7741	92.9%	(90.84, 95.16)
OPV3	7126	608	7734	92.1%	(90.03, 94.32)
All OPV^a^	7031	778	7809	90.0%	(88.07, 92.30)
All DPT/HepB/Hib^a^	7145	640	7785	91.8%	(89.72, 93.99)
Measles	6509	1093	7602	85.6%	(83.55, 87.73)

*BCG* Bacille-Calmette Guerin, *OPV* Oral Polio Vaccine, *DPT/HepB/Hib* Diphteria Pertussis Tetanus/Hepatitis B/*Haemophinlus influenzae* type b

^a^ All OPV or All DPT/HepB/Hib, refers to all doses of the vaccine having been correctly registered. It is lower than the last dose due to absence/incorrect documentation of some of the previous doses

^b^ Only eligible children (those who should have received a vaccine according to their age at the time of the visit and whose health card was assessed, readable and without missing dates) were included in this column

The multivariable logistic regression model revealed that children born in the municipality of Maluana had 89% higher odds of not receiving the vaccine compared to those born in central Manhiça (OR = 1.89, 95% CI: 1.18-3.00). Mothers’ marital status (divorced or not living with a male companion vs married or living with a male companion) showed a weak association with lack of vaccination: OR = 1.66 95% CI: 0.81-3.37) (Table 3). No other factors were associated with lack of BCG vaccination.

**Table 3:** Analysis of factors associated to lack of BCG vaccination

Variable	Number of children lacking BCG according to card	Total number of children with BCG informatio in the card	Bivariate analysis		Multivariable analysis	
			OR (95%CI)	*p*-value	OR (95%CI)	*p*-value
Sex						
Male	101 (2.6)	3932	1.0			
Female	102 (2.6)	3882	1.02 (0.77-1.35)	0.926		
Number of siblings						
None	0 (0)	9	–			
1 to 2	11 (1.3)	844	0.67 (0.31-1.44)			
3 or above	17 (1.9)	880	1.00	0.305		
Season of birth						
Rainy	108 (2.5)	4288	1.00			
Dry	96 (2.7)	3529	1.09 (0.82-1.44)	0.578		
Wealth Index						
1st Quintile	42 (3.2)	1312	1.00			
2nd Quintile	38 (2.6)	1478	0.82 (0.52-1.28)			
3rd Quintile	41 (2.8)	1450	0.90 (0.58-1.40)			
4th Quintile	28 (1.9)	1451	0.61 (0.37-0.99)			
5th Quintile	38 (2.7)	1418	0.85 (0.54-1.33)	0.325		
Administrative Post						
Manhiça Sede	37 (2.2)	1690	1.00		1.00	
3 de Fevereiro	34 (2.2)	1582	0.98 (0.61-1.57)		0.79 (0.45-1.39)	
Ilha Josina Machel	1 (0.7)	144	0.31 (0.04-2.29)		0.32 (0.04-2.39)	
Xinavane	57 (2.6)	2183	1.20 (0.79-1.82)		1.05 (0.59-1.89)	
Maluana	65 (3.9)	1671	1.78 (1.18-2.68)		1.89 (1.18-3.00)	
Calanga	10 (1.8)	547	0.83 (0.41-1.68)	0.007	0.47 (0.14-1.54)	0.003
Antenatal Visits						
1 to 2	3 (1.7)	168	1.00			
3 or above	25 (1.6)	1565	0.89 (0.37-2.98)	0.854		
Place of delivery						
Health centre	28 (1.7)	1655	1.00			
Home/way to hospital	0 (0)	77	–	–		
Type of delivery						
Natural	26 (1.6)	1645	1.00			
C-Section	2 (2.3)	88	1.44 (0.34-6.2)	0.618		
Mother’s marital status						
Single	12 (2.4)	510	1.00		1.00	
Married/Union	84 (2.2)	3868	0.92 (0.49-1.70)		0.99 (0.53-1.82)	
Divorced/Separated	23 (3.6)	631	1.57 (0.77-3.19)	0.083	1.66 (0.81-3.37)	0.092
Mother’s education						
No education	52 (2.5)	2089	1.00			
Primary	54 (2.4)	2284	0.95 (0.64-1.39)			
Secondary or higher	11 (2.3)	485	0.91 (0.47-1.75)	0.943		
Mother’s religion						
Christian	48 (2.3)	2104	1.00			
Muslim	1 (2.5)	40	1.09 (0.15-8.16)			
Traditional African	53 (2.5)	2158	1.07 (0.73-1.60)	0.902		
Others	8 (1.8)	428	0.82 (0.38-1.74)			
Distance to health centre						
Less than 5 km	17 (1.7)	983	1.00			
More than 5 km	88 (2.2)	3961	1.27 (0.75-2.15)	0.339		

### Scar assessment

From the 9512 adults who responded to the interview, irrespective of whether they presented the national health card or not, 8298 children could be directly observed for the presence of BCG-compatible scar. Coverage was 99.0% and 97.9% among children with and without a health card respectively. Therefore, when children are vaccinated with BCG (according to the health card), failure to develop the typical scar would occur in less than 1% in this population. We did not find any statistically significant association with lack of BCG scar. There were 174 children who were not BCG vaccinated according to the card, but 144 of them presented a BCG-compatible scar (82.8%).

### Timeliness of BCG

**Fig. 3:**
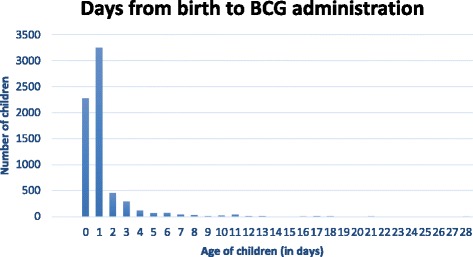
Timeliness of BCG administration

Figure 3 represents the distribution of BCG vaccines administered to children starting from the day of birth onwards. The results indicate that 93.4% of vaccinated children received BCG within the first 28 days of life. The factors associated with the administration of BCG in the first 28 days of life are described in Table 4. The only factor associated with a timely BCG administration is not being born by a cesarean section (for which OR = 0.40, *p*-value 0.021). In other words, children born through a cesarean section are 60% less likely to have an adequate administration of BCG vaccine.

**Table 4:** Analysis of factors associated to a adequate timeliness of BCG administration (within 28 first days of life) according to health card

Variable	Timely BCG vaccinated (%)	Total children with BCG	Bivariate analysis		Multivariable analysis	
			OR (95%CI)	*p*-value	OR (95%CI)	*p*-value
Sex						
Male	3449 (93.4)	3691	1.00			
Female	3399 (93.3)	3642	0.98 (0.82-1.18)	0.842		
Number of siblings						
None	9 (100)	9	1.00			
1 to 2	750 (95.4)	786	1.25 (0.80-1.95)			
3 or above	769 (94.4)	815	1.00	0.335		
Season of birth						
Rainy	3743 (93.3)	4012	1.00			
Dry	3108 (93.5)	3324	1.03 (0.86-1.24)	0.723		
Wealth Index						
1st Quintile	1136 (93.3)	1218	1.00			
2nd Quintile	1289 (93.5)	1378	1.05 (0.77-1.42)			
3rd Quintile	1265 (93.1)	1359	0.97 (0.71-1.32)			
4th Quintile	1278 (93.9)	1361	1.11 (0.81-1.52)			
5th Quintile	1260 (94.3)	1336	1.19 (0.86-1.65)	0.704		
Administrative Post						
Manhiça Sede	1496 (94.6)	1582	1.00			
3 de Fevereiro	1364 (93.0)	1466	0.77 (0.57-1.03)			
Ilha Josina Machel	123 (94.6)	130	1.01 (0.46-2.23)			
Xinavane	1941 (93.5)	2077	0.82 (0.62-1.08)			
Maluana	1431 (91.7)	1560	0.64 (0.48-0.85)			
Calanga	496 (95.2)	521	1.14 (0.72-1.80)	0.018		
Antenatal Visits						
1 to 2	148 (95.5)	155	1.00			
3 or above	1380 (94.8)	1455	0.87 (0.39-1.92)	0.731		
Place of delivery						
Health centre	1458 (95.0)	1535	1.00			
Home/way to hospital	69 (93.2)	74	0.73 (0.29-1.86)	0.507		
Type of delivery						
Natural	1453 (95.2)	1527	1.00		1.00	
C-Section	75 (90.4)	83	0.48 (0.22-1.03)	0.058	0.40 (0.18-0.87)	0.021
Mother’s marital status						
Single	424 (90.0)	471	1.00		1.00	
Married/Union	3421 (93.8)	3649	1.66 (1.19-2.31)		1.50 (0.76-2.94)	
Divorced/Separated	544 (94.4)	576	1.88 (1.18-3.00)	0.006	1.50 (0.56-4.00)	0.490
Mother’s education						
No education	1836 (93.1)	1973	1.00			
Primary	1994 (93.6)	2131	1.09 (0.85-1.39)			
Secondary or higher	430 (74.7)	576	1.33 (0.86-2.09)	0.421		
Mother’s religion						
Christian	1834 (93.4)	1964	1.00			
Muslim	35 (89.7)	39	0.62 (0.22-1.77)			
Traditional African	1907 (94.2)	2025	1.15 (0.89-1.48)			
Others	376 (93.3)	403	0.99 (0.64-1.52)	0.528		
Distance to health centre						
Less than 5 km	61 (72.6)	84	1.00		1.00	
More than 5 km	205 (5.9)	3495	1.24 (0.92-1.66)	0.161	1.48 (0.90-2.44)	0.118

## Discussion

### Main findings

This study provides population estimates of BCG administration by two different methods in a large cohort of children. It shows that vaccine coverage in Manhiça district was very high for all vaccines administered in the first year of life, surpassing the international targets for EPI vaccine coverage. This finding is in line with results presented from similar studies about EPI vaccine coverage in Mozambique [29].

This is the first vaccination coverage study in the country using data collected by a HDSS. This preliminary information could be very relevant for future vaccine trials and a proxy for other health interventions. It is also important to highlight the importance of data registries in LMIC to monitor health systems’ performance, resource allocation planning and progress in immunization strategies. These findings call for an improved system to collect information to be used for assessing vaccine coverage, and which could hopefully be used to compare across different countries.

In the period from 2011 to 2014, BCG coverage was 97.4%, higher than the estimation of 86.3% in Maringue District, Sofala Province (centre Mozambique) [12] and the nationwide 94% estimation by WHO [28]. The results of high coverage could be explained because of the likely better health infrastructure in the district than national standards, which include two referral hospitals plus the existence of a research centre (the CISM, which conducts operational and translational research). The latter, conducts at least one visit per year to each household for the purpose of HDSS work rounds of data collection in the district, which could potentially affect vaccination-seeking behaviour in the community. However, selection bias might have occurred since there is a proportion of subjects who fail to provide a health card. Although the main stated reason was that the caregivers could not find the card, if those who did not find the card had lower vaccine coverage, our estimates might represent a slight overestimation of the true coverage. An extra source of potential selection bias is that those born in that period who died before the HDSS census rounds might have had lower BCG coverage. However, the effect of this bias, albeit unknown, could be limited, since those with and without vaccination card had similar coverages measured by the presence of scar.

We found no statistically significant associations with lack of BCG vaccination, except living in the municipality of Maluana. These findings might be explained by the small number of non-vaccinated individuals (random error) or other social factors that will require qualitative approaches in order to be identified.

Very few BCG vaccinated children (according to their health cards) in the district of Manhiça fail to develop the scar. These results are comparable with findings of scar failure in other countries, ranging from 1 to 20% [13, 38, 39]. Potential observer bias could have taken place, since field workers were not blind to the child heath card information. Nonetheless, the proportion of scar formation in children with and without health card was similar to that of BCG vaccinated infants. If these findings were a true overestimation, the reason behind could be a systematic poor evaluation of the presence of BCG scar. Conversely, the fact that many children with no record of BCG in their card presented BCG scar could lead to a potential underestimation of coverage estimates based solely on immunization card. This could be due to bad documentation of BCG vaccination in the immunization card (or cases where the card was lost and replaced, and information could not be updated). Unfortunately these potential explanations cannot be verified.

Recent studies showing scar beneficial effects, such as lower mortality in infants with scar [5, 11], have opened the debate about re-vaccination [5, 40] among those failing to develop a scar. Some have suggested that scarring could be a method to monitor vaccination performance in resource-poor settings. On the other hand, BCG is not recommended in HIV suspected cases and HIV-related immunosuppression may play a role in scar response. In a high HIV burden country such as Mozambique, where most children are BCG vaccinated regardless of their HIV status, we expected a lower scar formation rate.

The timing of vaccination is very important in order to reach the maximum protection, but also for being a proxy of non-adherence and reduce of vaccination. [16, 17, 20, 22]. In order to measure if BCG was appropriately administered, we consider a timely vaccination if it occurred within the 28 days of life, as recommended by WHO [19]. The results show a low proportion of delayed BCG vaccination (6.6%), compared to 33% found in Tanzanian the year 2004 [15]. However, the definition of delayed BCG vaccination differs from author to author, [19] some consider it happens only after 8 weeks or even after 56 days [17] after birth, thus comparability with other studies needs to be cautious. The only factor associated with timely BCG vaccination was being delivered through a caesarean section. It is closely related with being born in a health facility, with a skilled birth attendant, where they will have the BCG vaccine ready to be administered after birth.

This study had several limitations. First, selection bias could have occured since we could only visit children whose adults were present at the moment of the interview and presented the card (for evaluation of the registration) and/or the children were present (for scar assessment). There were 16.9% (1609/9512) of children who did not present a health card. Although most of them argued that adults had lost the card, these children might live in families with more difficulties in accessing the health system or not able to have a proper follow-up of their children’s health status, thus our vaccination coverage could be overestimating the real one. Secondly, children who died before the first round visit were not included and might have different (potentially lower) vaccine coverage. Thirdly, given the discrepancies found about BCG vaccination assessed through health card and presence of scar, poor BCG documentation in the card or poor evaluation of BCG scar, cannot be ruled out. Last, due to the low number of non-vaccinated individuals identified, the study had little power to detect potential factors associated with absence of vaccination.

## Conclusions

This study shows high vaccination coverage in Manhiça district; although vaccines that need several doses or that are administered months after birth require larger efforts to ensure all children are properly and completely vaccinated. The vast majority of BCG vaccines are given within the first days after birth. Scar development occurs in almost all infants. No associations with lack of BCG were found, except for living in the municipality of Maluana. These findings require targeted investigations to find out potential reasons for that difference in coverage that might benefit from tailored interventions. Prospective data collection at the time of vaccination would avoid potential bias inherent to retrospective data collection. This research study, beyond high coverage of BCG and other EPI vaccines, shows the importance of having data registries in LMIC to monitor health systems’ performance, resource allocation planning and progress in immunization strategies.

## Additional file

Additional file 1: Demographic and socioeconomic characteristics of less than 36-months old children with and without card. In this table we expand the baseline demographic and socioeconomic characteristics of study participants depending on the availability of the health card. (DOCX 19 kb)

## Abbreviations

AIDS: Acquired immune deficiency syndrome
BCG: Bacille Calmette-Guérin
CI: Confidence interval
CISM: Centro de Investigação em Saúde de Manhiça
DPT/HepB/hib: Diphtheria Pertussis Tetanus/Hepatitis B/*Haemophilus influenza* type b (pentavalent vaccine)
DSS: Demographic surveillance system
EPI: Expanded programme on immunization
HDSS: Health and demographic surveillance system
HIV: Human immunodeficiency virus
LIC: Low income Countries
LMIC: Low and middle income Countries
OPV: Oral polio vaccine
OR: Odds ratio
TB: Tuberculosis
TST: Tuberculin skin test
WHO: World Health Organization
