# Supplementary material for: BCG vaccination in southern rural Mozambique: an overview of coverage and its determinants based on data from the demographic and health surveillance system in the district of Manhiça
Source: BMC Pediatr. 2018 Feb 13;18:56. doi: 10.1186/s12887-018-1003-4 (PMC5811981; doi:10.1186/s12887-018-1003-4)
Supplement: Supplementary file 1 — Demographic and socioeconomic characteristics of less than 36-months old children with and without card. In this table we expand the baseline demographic and socioeconomic characteristics of study participants depending on the availability of the health card. (DOCX 19 kb) [file 12887_2018_1003_MOESM1_ESM.docx]

Additional file 1.

|  |
| --- |
